# Lithium inhibits tumor lymphangiogenesis and metastasis through the inhibition of TGFBIp expression in cancer cells

**DOI:** 10.1038/srep20739

**Published:** 2016-02-09

**Authors:** Yong-Sun Maeng, Rina Lee, Boram Lee, Seung-il Choi, Eung Kweon Kim

**Affiliations:** 1 Department of Ophthalmology, Corneal Dystrophy Research Institute, Yonsei University College of Medicine, Seoul, South Korea; 2 Institute of Vision Research, Severance Biomedical Science Institute, Brain Korea 21 Plus Project for Medical Science, Yonsei University College of Medicine, Seoul, South Korea

## Abstract

Metastasis is the main cause of mortality in cancer patients. Although there are many anti-cancer drugs targeting tumor growth, anti-metastatic agents are rarely developed. Angiogenesis and lymphangiogenesis are crucial for cancer progression; in particular, lymphangiogenesis is pivotal for metastasis in cancer. Here we report that lithium inhibits colon cancer metastasis by blocking lymphangiogenesis. Lithium reduces the expression of transforming growth factor-β-induced protein (TGFBIp) in colon cancer cells by inhibiting Smad3 phosphorylation via GSK3β inactivation. Moreover, lithium inhibits lymphatic endothelial cell migration, which is increased upon TGFBIp expression in tumor cells. Lithium had no significant effect on SW620 tumor growth *in vitro* and *in vivo*; however, it inhibited lymphangiogenesis in tumors. In tumor xenografts model, lithium was found to prevent metastasis to the lungs, liver, and lymph nodes by inhibiting TGFBIp-induced tumor lymphangiogenesis. Collectively, our findings demonstrate a novel role of lithium in the inhibition of colon cancer metastasis by blocking TGFBIp expression, and thereby TGFBIp-induced lymphangiogenesis, in primary tumors.

Cancer metastasis follows an ordered and hierarchical pattern. Colorectal cancer cells initially spread to the lymph nodes and peritoneal area. When metastases to the liver occur, a substantial number of patients also develop lung, and less frequently bone or brain, metastases^1^. Patients with metastasis are treated with systemic chemotherapy, mostly in a palliative manner. Nevertheless, in selected patients with isolated liver metastasis, increased 5-year survival can be achieved by multimodal treatment that includes a combination of surgery with modern chemotherapy^2^. However, only about 25% of patients can benefit from this type of treatment, and the presence of metastases in other organs is, in most cases, a contraindication for resection^3^. Although metastatic tumor spread can occur via a variety of mechanisms, including direct local invasion of tissue or the seeding of body cavities, most metastases arise following invasion of, and dissemination via, the circulatory system.

Although anti-angiogenic agents have been used for treating cancer in the clinical setting, overall survival in advanced cancer patients has not been substantially improved^4^, possibly because cancer metastasis occurs preferentially via lymphatic dissemination rather than hematogenous spread^5^,^6^,^7^. Indeed, in many human cancers, the detection of tumor metastases in the tumor-draining lymph node is the first step in tumor dissemination, and is one of the most important markers of both patient prognosis and therapeutic strategy decisions. Thus, there is an unmet need to develop therapeutic agents to block lymphangiogenesis as well as angiogenesis, in order to efficiently block tumor growth and metastasis.

Using bioinformatics-aided methodologies and *in vitro* and animal experiments, we have identified an extracellular matrix protein that is involved in lymphangiogenesis^8^. We have reported that transforming growth factor-β-induced protein (TGFBIp) derived from cancer cells or lymphatic endothelial cells promotes lymphatic endothelial cell (LEC) migration, adhesion, and tube formation^8^. We have also documented that TGFBIp derived from colon cancer cells exhibits potent pro-lymphangiogenic activity in tumor xenografts^8^. Furthermore, we have shown that the inhibition of TGFBIp expression in cancer cells using the TGFBIp shRNA system decreases tumor lymphangiogenesis and metastasis to distant organs^8^. However, TGFBIp had a minor effect on migration, tube formation, and sprouting of human umbilical vein endothelial cells, and the inhibition of TGFBIp expression showed only weak anti-angiogenic activity^8^. When tested separately, the anti-lymphangiogenic effect of inhibition of TGFBIp expression was significantly more potent than its anti-angiogenic activity in primary tumors^8^. Based on these findings, we suggested that TGFBIp exerts a stronger effect on lymphatic vessels than on blood vessels, and further that TGFBIp is a potential target to block tumor lymphangiogenesis and metastasis^8^.

Recently, several studies have revealed that GSK-3 inhibitors downregulate TGFBIp expression by blocking TGF-β signaling^9^,^10^,^11^. GSK-3 inactivation generally yields anti-apoptotic effects. A number of studies demonstrate that lithium directly inhibits GSK-3^12^,^13^,^14^,^15^,^16^,^17^. Lithium also indirectly inhibits GSK-3 by triggering the phosphorylation of GSK-3 at ser21/ser9^18^,^19^,^20^. Increasing evidence suggests that lithium elicits its neuroprotective effects by inhibiting GSK-3^21^. Besides direct inhibition, lithium can block GSK-3 activity indirectly through the phosphorylation of GSK-3α at ser21 and of GSK-3β at ser9 by multiple mechanisms, including the activation of PKA^22^, phosphatidylinositol 3-kinase (PI3-K)-dependent AKT^18^, and protein kinase C (PKC)^23^, and autoregulation of GSK-3^20^,^24^. One of our recent studies also demonstrated that lithium treatment reduces TGFBIp expression in a dose-dependent manner in corneal fibroblasts through the inactivation of GSK-3^25^. Therefore, we investigated the effects of lithium on TGFBIp expression and lymphangiogenesis in colon cancer cells.

Here, we report the *in vitro* and *in vivo* activities of lithium in inhibiting TGFBIp expression, tumor lymphangiogenesis, and metastasis. Lithium reduces the expression of TGFBIp in SW620 colon cancer cells by inhibiting the transforming growth factor β1-Smad3 signaling pathway via GSK3β inactivation. In addition, lithium inhibits lymphatic endothelial cell (LEC) migration induced by TGFBIp. Furthermore, we demonstrated that lithium has activity against lymphangiogenesis and angiogenesis, has no effect on the growth of a primary colon cancer tumor xenograft, and strongly inhibits its metastasis to the lungs, liver, and lymph nodes by blocking lymphangiogenesis in primary tumors. Taken together, these data suggest that lithium functions as an anti-tumor metastasis factor by inhibiting TGFBIp expression and TGFBIp-induced tumor lymphangiogenesis in primary tumors.

## Results

### Lithium inhibits TGFBIp expression in tumor cells

To assess the effect of lithium on TGFBIp expression in SW620 colon cancer cells, which highly expressed the TGFBIp protein^8^,^26^, colon cancer cells were cultured with lithium carbonate, and then TGFBIp expression was analyzed. The concentration of lithium carbonate used to treat cancer cells was determined empirically. Usually, high lithium doses, over 20 mM, are used for their effect on cancer^27^,^28^. However, high lithium doses did not affect TGFBIp expression in our experimental condition and low lithium doses (125–2000 μM) only reduced of TGFBIp expression in cancer cells. Cancer cells cultured with lithium carbonate displayed significantly decreased TGFBIp protein and mRNA levels, and this effect was dose-dependent (Supplementary Fig. S1a, b online). Moreover, lithium carbonate inhibited TGFβ1-induced TGFBIp expression in a dose-dependent manner (Supplementary Fig. S1c online).

The decrease in TGFBIp protein levels in response to lithium might occur via one of two mechanisms: lithium may enhance TGFBIp degradation or decrease TGFBIp biosynthesis. We previously demonstrated that autophagy is the main intracellular degradation pathway for TGFBIp and that lithium activates it through the PI3K signaling pathway^25^,^29^. We also provided evidence indicating that the activation of autophagy enhances the cytosolic clearance of intracellular TGFBIp in corneal fibroblasts^29^. Therefore, we investigated the effect of lithium on autophagy in cancer cells. However, lithium did not induce TGFBIp degradation via the autophagy-lysosome system (unpublished data).

The identification of Smad3/GSK-3 complexes^30^ suggests that GSK-3 can activate Smad3, a component of the TGFβ1 signaling pathway; moreover, lithium is a well-established GSK-3 inhibitor. Therefore, we examined the effects of lithium on TGFBIp expression by investigating the TGFβ1 signaling pathway. As shown in Supplementary Fig. S1d online, lithium carbonate decreased Smad3 phosphorylation in a dose-dependent manner. In addition, lithium carbonate interacts with GSK-3α/β and negatively regulates its activity. Treatment of cancer cells with lithium carbonate significantly increased GSK-3β phosphorylation (Supplementary Fig. S1d online). Taken together, these results suggest that lithium reduces the expression of TGFBIp in colon cancer cells by inhibiting the TGFβ1-Smad3 signaling pathway via GSK3ββ inactivation.

### Lithium had no effect on tumor growth

**Figure 1:**
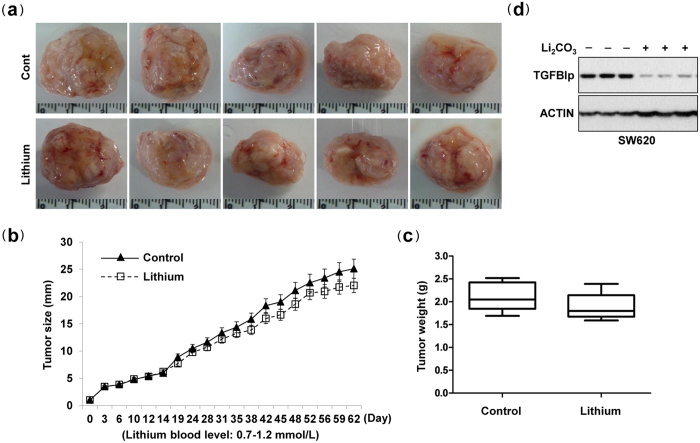
Lithium carbonate had no effect on tumor growth. SW620 cells were injected subcutaneously into the midline of the backs of NOS-SCID mice (n = 5 per group). After tumor cell inoculation, mice were randomized to receive either 125 mg/kg lithium carbonate (Li_2_CO_3_) in 100 μL deionized water (d.H_2_O), or 100 μL d.H_2_O alone, two times per week by oral gavage, from the time of tumor inoculation. Tumors were removed after 62 days, excised, and serially sectioned. (**a**) Representative tumors. (**b**,**c**) Analysis of tumor growth rates and weights. (**d**) TGFBIp protein levels in tumors derived from lithium- or control-injected mice were measured by western blotting.

We evaluated the capacity of lithium to inhibit TGFBIp-induced tumor growth, angiogenesis, lymphangiogenesis, and metastasis in an animal model: a subcutaneous xenograft of the human colon cancer SW620 cell line that displays an aggressive metastatic pattern^8^,^26^. After tumor injection (5 × 10^6^ cells/mouse), lithium carbonate was administered orally two times per week for up to 62 days (Supplementary Fig. S2 online). Equal numbers of SW620 cells were implanted subcutaneously into NOD-SCID mice, and the growth of the resultant primary tumors was monitored. Interestingly, there were no significant differences in the average size and weight of the tumors derived from control and lithium carbonate-treated mice (Fig. 1a–c). Mouse body weights were statistically identical among the two groups, demonstrating that lithium carbonate does not have severe toxic effects (unpublished data). Additional western blot assays showed that TGFBIp protein levels were significantly reduced in tumors derived from lithium carbonate-treated mice, compared with those from controls (Fig. 1d). Additionally, *in vitro* growth rates were not modified by lithium treatment (Supplementary Fig. S3 online). These data demonstrate that lithium had no significant effect on tumor growth *in vitro* and *in vivo*.

### Lithium reduces tumor lymphangiogenesis

**Figure 2:**
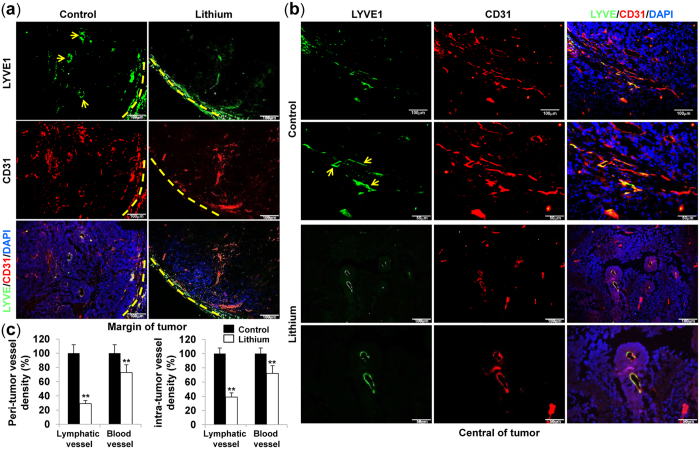
Lithium-carbonate reduces tumor lymphangiogenesis in colon cancer. (**a**) Margins of tumor sections were stained for infiltrating LECs using anti-LYVE1 and anti-CD31 antibodies. Nuclei were stained with DAPI (blue fluorescence). Arrows indicate lymphatic vessels. Yellow dotted lines point to the margins of the tumor. Images were viewed using an Olympus IX81-ZDC microscope with a LUCPL FLN 10×/1.0 NA lens. (**b**) Central regions of tumor sections were stained for infiltrating LECs using anti-LYVE1 and anti-CD31 antibodies. Nuclei were stained with DAPI (blue fluorescence). Arrows indicate lymphatic vessels. Images were viewed by using an Olympus IX81-ZDC microscope with a LUCPL FLN 10×/1.0 or 2.0 NA lens. **(c**) Quantitative assessment of LYVE1- and CD31-positive lymphatic vessels per field for each tumor section. Data are presented as the mean ± S.E. **P < 0.01 *vs*. control group.

To determine whether lithium could inhibit tumor angiogenesis and lymphangiogenesis, tumors were sectioned and stained for lymphatic- or blood vessel-specific markers, LYVE-1 and CD31, respectively. Tumors derived from lithium-treated mice showed a very low density of lymphatic vessels stained with LYVE-1/CD31 in the peri-tumoral (margin of tumor) (Fig. 2a) and intratumoral (central region of tumor) areas (Fig. 2b and Supplementary Fig. S4 online). Quantification of LYVE-1/CD31-positive lymphatic structures confirmed that vessel density was significantly reduced in lithium-treated tumors as compared with controls (Fig. 2c). However, only CD31-positive blood vessel density was slightly decreased in lithium-treated tumors (Fig. 2c).

To further validate the effect of lithium on tumor lymphangiogenesis by TGFBIp expressed in tumor cells, a Transwell migration assay was performed. As shown in Supplementary Fig. S5 online, tumor cell increased the migration of LECs, which were used as a positive control. However, lithium-treated tumor cells showed decreased migration of LECs, and this migration was re-increased by TGFBIp treatment. These results indicate that lithium reduces tumor (lymph)angiogenesis by inhibiting TGFBIp expression in tumor cells *in vitro* and *in vivo*.

### Lithium inhibits tumor metastasis

**Figure 3:**
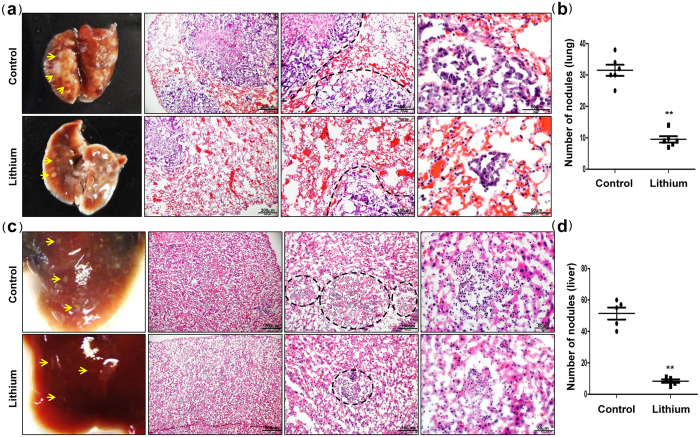
Lithium carbonate suppresses tumor metastases to lung and liver. **(a**,**c**) Images of lung and liver metastases of mice injected with SW620 cells, and hematoxylin and eosin staining of frozen sections of lung and liver tissues isolated from mice subcutaneously injected with SW620 cells. (**b**,**d**) The number of metastatic nodules per lung or liver was quantified by microscopic inspection. **P < 0.01 *vs.* control group. Black dotted lines indicate metastatic tumors in the mouse lung or liver. Data are presented as the mean ± s.e. **P < 0.01 *vs.* control group.

**Figure 4:**
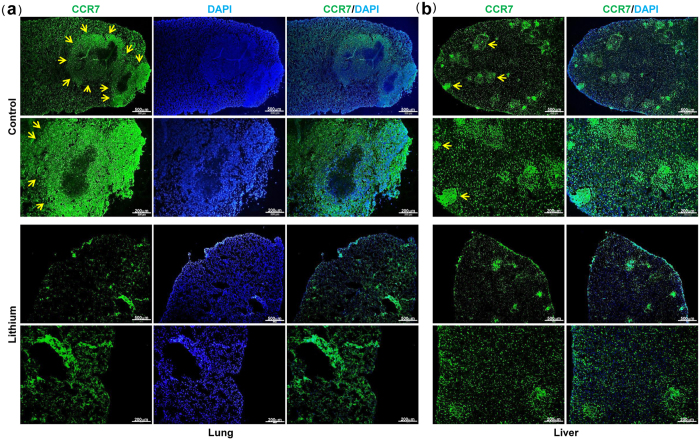
Lithium carbonate suppresses CCR7^+^ tumor metastases to lung and liver. (**a**,**b**) CCR7 staining of frozen sections of lung and liver tissues isolated from mice subcutaneously injected with SW620 cells. Arrows indicate CCR7-positive metastatic tumors in the mouse lung or liver.

**Figure 5:**
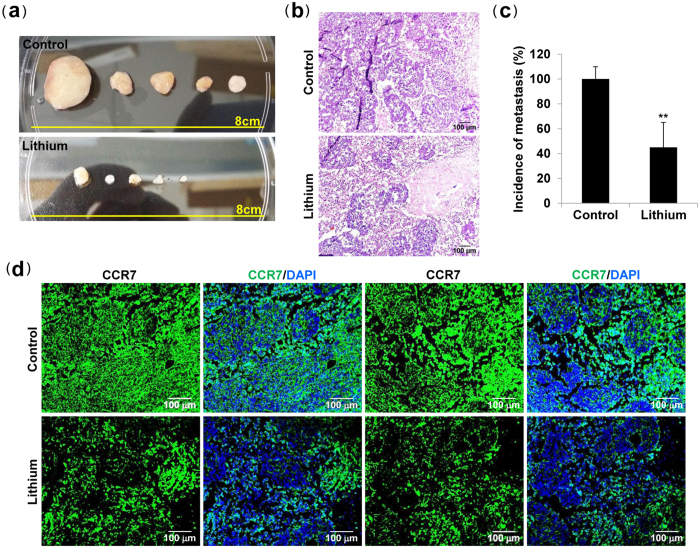
Lithium carbonate suppresses tumor metastasis to the lymph nodes. (**a**) Lateral axillary lymph node morphology of tumor-bearing groups (n = 5 per group). (**b**,**d**) Hematoxylin and eosin, and CCR7 staining of frozen sections of lateral axillary lymph node tissues isolated from mice subcutaneously injected with SW620 cells. (**c**) The incidence of metastasis per lateral axillary lymph node was quantified by microscopic analysis. Data are presented as the mean ± s.e. **P < 0.01 *vs*. control group.

Finally, to evaluate the effects of lithium on tumor metastasis via the lymphatic system, tumor-bearing mice were sacrificed and examined for metastases in the lung and liver. Microscopic analysis of the lungs and livers revealed a significant decrease in the number of metastatic nodules per lung or liver from lithium-treated mice versus control mice (Fig. 3a,c), which was confirmed by quantification of the number of metastatic nodules per lung or liver (Fig. 3b,d). Moreover, immunohistochemical analysis and tumor cell-specific CCR7 staining revealed significantly reduced lung (Figs 3a, 4a and Supplementary Fig. S6a online) and liver metastases (Figs 3c, 4b and Supplementary Fig. S6b online) in lithium-treated mice, compared with control mice. Strikingly, lithium treatment inhibits the incidence of lateral axillary lymph node metastasis, and histological analysis showed reduced metastatic colon cancer cells in the lymph node in lithium-treated mice versus control mice (Fig. 5a–d and Supplementary Fig. S7 online). Overall, our data suggest that lithium treatment is useful to suppress the metastatic potential of colon cancer cells via the lymphatic system without affecting the growth rate of tumors.

## Discussion

Generally, after the removal of malignant tumors by surgery, radiation therapy and/or adjuvant treatment with chemotherapy may be curative. However, the removal of certain cancers, for example breast carcinomas, colon carcinomas, and osteogenic sarcomas, may be followed by rapid metastasis to the lung, liver, and kidney through neovascularization and/or to regional lymph nodes through the lymphatic system^6^,^7^,^31^,^32^,^33^,^34^,^35^,^36^,^37^. Therefore, it is necessary to develop new anticancer agents with anti-tumor and anti-metastatic activities. Several reports have shown that lithium is effective in inhibiting glioma^38^, colorectal cancer^39^, medulloblastoma^40^, hepatocellular carcinoma^41^, and other cancer cells^42^. In fact, it has been suggested that the lower cancer prevalence observed in mental patients is likely due to a protective benefit derived from lithium treatment^43^. At the molecular level, lithium has been shown to inhibit the growth and/or tumorigenicity of cancer cells by modulating the biological activities of many cancer-related genes, such as STAT3^44^, b-catenin/WNT^39^,^40^, TNF^42^, FASL^45^ and P53^46^. Lithium, an effective GSK3β inhibitor, has been used in treating depression and bipolar disorder for many years^47^. Recently, it has been shown that inhibition of GSK3β promotes apoptosis in glioma cells and PDA cells^48^,^49^, and sensitizes PANC-1 cells to gemcitabine^50^. In addition, lithium induces apoptosis in a variety of cancer cells^45^,^51^. At present, the mechanism of lithium-mediated anti-cancer activity is not clear, and the anti-metastatic actions of lithium have yet to be demonstrated *in vitro* and *in vivo*.

We previously reported that TGFBIp is a lymphangiogenic factor that induces metastasis via three probable mechanisms: (1) by increasing lymphatic density and consequently augmenting surface contact with cancer cells; (2) by increasing CCL21 expression in LECs and promoting the migration of cancer cells; and (3) by increasing lymphatic permeability and facilitating the intravasation and/or extravasation of tumor cells^8^ (Supplementary Fig. S8 online). Based on these findings, we suggested that TGFBIp might be a potential therapeutic target for treating metastatic colon cancer. Inhibition of TGFBIp expression using a TGFBIp-specific shRNA lentivirus in tumor cells or LECs dramatically reduced adhesion, migration, lymphatic sprouting of LECs, and tumor lymphangiogenesis and metastasis *in vitro* and *in vivo*. These finding suggest that inhibition of TGFBIp expression helps suppress the metastatic potential of colon cancer cells via owing to the consequent reduced lymphangiogenesis^8^.

In this study, we investigated the inhibitory effect of lithium on TGFBIp expression, tumor lymphangiogenesis, and metastasis *in vitro* and *in vivo*. Interestingly, our results show a significant downregulation of TGFBIp expression in colon cancer cells following lithium treatment, and further demonstrate that lithium induces this effect by inhibiting Smad3 phosphorylation though GSK3β inactivation. Several studies indicate that GSK-3 inhibitors downregulate TGFBIp expression by blocking TGFβ signaling^9^,^10^,^11^. To test this hypothesis, we analyzed the expression of TGFBIp in cancer cells treated with lithium. Western blot analysis showed that lithium treatment reduced the levels of TGFBIp in a dose-dependent manner. Quantitative RT-PCR indicated that lithium treatment decreased TGFBIp mRNA expression in a dose-dependent manner, suggesting that the regulation of TGFBIp occurs at the transcriptional level. In addition, we analyzed whether lithium could inhibit TGFβ1-SMAD signaling by blocking GSK-3 activity. We found that lithium negatively regulated GSK-3 activity and decreased Smad3 phosphorylation, thereby reducing TGFBIp expression in a dose-dependent manner. However, lithium did not induce TGFBIp degradation via the autophagy-lysosomal system, the main intracellular degradation pathway for TGFBp^29^. Collectively, these results suggest that lithium reduces the expression of TGFBIp in cancer cells by inhibiting the TGFβ1-Smad3 signaling pathway through the inactivation of GSK3β.

In addition, TGFBIp expression in tumors of mice with subcutaneously implanted SW620 colon cancer cells was significantly reduced in lithium-treated mice compared with control mice. Interestingly, there was no significant difference in the average size and weight of tumors in control and lithium-treated mice, and the *in vitro* growth rates were not influenced by lithium treatment. These results suggest that low lithium doses in our experimental conditions only reduce TGFBIp expression but may not be sufficient to inhibit proliferation or induce apoptosis in cancer cells. Usually, high lithium doses affect cancer proliferation or apoptosis^27^,^28^. In addition, the effect of lithium on tumor growth may not only dependent on the inhibition of TGFBIp expression, but also on the regulation of oncogenes such as STAT3,β-catenin/WNT, TNF, FASL, and P53. This possibility needs to be investigated in further studies.

Inhibition of TGFBIp expression in cancer cells by lithium decreased tumor metastasis to the lungs, liver, and lymph nodes. Based on immunohistochemical observations, lithium strongly reduced the number of lymphatic vessels (LYVE-1/CD31-positive cells) in the peri- and intra-tumoral areas. However, the density of blood vessels that were only CD31-positive was slightly decreased in lithium-treated tumors, suggesting that lithium has a stronger effect on lymphatic vessels than on blood vessels. Lymphatic vessels with wider lumens were observed in control tumors expressing high levels of TGFBIp, and were correlated with increased tumor metastasis to the lungs, liver, and lymph nodes. To further evaluate the effect of lithium on tumor lymphangiogenesis by TGFBIp expressed in tumor cells, an *in vitro* transwell migration assay was performed. Tumor cells expressing high levels of TGFBIp showed increased LEC migration. However, lithium-treated tumor cells showed decreased LEC migration, and this migration was increased by TGFBIp treatment. These results demonstrate that lithium inhibits tumor (lymph)angiogenesis by inhibiting TGFBIp expression in tumor cells *in vitro* and *in vivo*.

Furthermore, analysis of tumor cell metastasis in mouse organs revealed a significant decrease in the number of metastatic nodules per lung or liver, as well as reduced lateral axillary lymph node metastasis, in lithium-treated mice as compared with control mice. Our data suggest that the inhibition of TGFBIp by lithium administration is useful to suppress the metastatic potential of colon cancer cells via the lymphatic system.

The anti-metastatic activity of lithium provides clinical insights and a rationale for using lithium as an agent to prevent metastasis in cancer patients in neoadjuvant settings. In the neoadjuvant setting, where the tumor is not excised during chemotherapy, the stressed tumor may secrete factors to promote angiogenesis and lymphangiogenesis at distant sites, in order to allow it to move to a more favorable site where the local tumor microenvironment may protect it. If such patients with cancer cells expressing high levels of TGFBIp are treated with lithium, possibly in combination with chemotherapeutics, metastatic lymphatic vessel formation would be inhibited. As a result, secondary recurrence of the tumor, including metastases, may be more effectively prevented.

In summary, our findings clearly demonstrate a novel role of lithium to inhibit colon cancer metastasis by blocking TGFBIp expression, and thereby TGFBIp-induced lymphangiogenesis, in primary tumors (Supplementary Fig. S8 online). As lymphangiogenesis is central to metastatic cell dissemination and metastatic disease is the primary cause of death in cancer, lithium could be a potential treatment for metastatic disease.

## Methods

### Cell culture

SW620 cells were purchased from the Korean Cell Line bank (Seoul, Korea) and cultured in Dulbecco’s modified Eagle medium (DMEM; Invitrogen, Carlsbad, CA) supplemented with 10% fetal bovine serum (FBS) and antibiotics.

### Cell migration assay

Cell migration was assayed using the Transwell system (Corning Costar, Acton, MA) with 6.5 mm-diameter polycarbonate filters (8-μm pore size). Briefly, (group 1, 2) the lower surface of the filter was coated with 10 μg/ml gelatin. LECs (10^5^) were seeded onto chemotaxis filters, and recombinant human TGFBIp (rhTGFBIp; 10 μg/ml; Sino Biological Inc., Beijing, China) was then added to the lower chamber. (group 3, 4) the lower surface of the filter was coated with 10 μg/ml gelatin. SW620 tumor cells were seeded in the lower chamber until confluence and were either treated with TGFBIp (10 μg/ml) or not. LECs (10^5^) were seeded onto chemotaxis filters. (group 5) the lower surface of the filter was coated with 10 μg/ml gelatin. SW620 tumor cells were seeded in the lower chamber until confluence and treated with lithium carbonate (2000 μM) for 60 min. After a change in media, LECs (10^5^) were seeded onto chemotaxis filters. (group 6) the lower surface of the filter was coated with 10 μg/ml gelatin. SW620 tumor cells were seeded in the lower chamber to confluence and treated with or without lithium carbonate (2000 μM) for 60 min. After a change of media, TGFBIp (10 μg/ml) was then added to the lower chamber. LECs (10^5^) were seeded onto chemotaxis filters. After the 12-hour migration period, non-migrating cells were completely removed from the top surface of the membrane. Migrating cells adhering to the undersurface of the filters were detected by hematoxylin and eosin (H&E) staining and quantified using Kodak 1D software (Eastman Kodak, Rochester, NY). Results are representative of three different experiments, each performed in duplicate.

### Real-time quantitative reverse transcription polymerase chain reaction (Real-time qRT-PCR)

Total RNA was isolated from SW620 cells by extraction in TRIZOL reagent (Invitrogen, Carlsbad, CA). Using the Power SYBR Green RNA-to-CT^TM^ 1-Step kit (Applied Biosystems, Foster City, CA) and StepOnePlus^TM^ (Applied Biosystems), mRNA expression levels of the human GAPDH and TGFBIp genes were measured according to the manufacturer’s instructions. The PCR conditions for all genes were as follows: 48 °C for 30 min, 95 °C for 10 min, then 40 cycles of 95 °C for 15 s and 60 °C for 1 min. The results are based on cycle threshold (Ct) values. We calculated differences between the Ct values for experimental and reference genes (GAPDH) and graphed the results as the ratio of each RNA to the calibrator sample. The primers used for gene amplification were as follows: TGFBI 5′-CACAGTCTTTGCTCCCACAA-3′ (sense) and 5′-CTCCGCTAACCAGGATTTCA-3′. (antisense); GAPDH 5′-ATGGGGAAGGTGAAGGTCG-3′ (sense) and 5′-GGGGTCATTGATGGCAACAATA-3′ (antisense). Three independent experiments were performed, and statistical analysis was carried out using Newman-Keuls multiple comparison tests.

### Immunofluorescence staining and immunohistochemistry

Eight micrometer-thick frozen tumor sections were washed in phosphate-buffered saline (PBS) and blocked in 5% normal donkey serum in an antibody dilution buffer consisting of PBS containing 0.1% Triton X-100. Sections were then incubated overnight in primary antibody (rat anti-mouse CD31 monoclonal antibody; Clone: MEC 13.3, 1:200 dilution, BD PharMingen, San Diego, CA) or rabbit anti-mouse LYVE1 polyclonal antibody (1:200 dilution, Angiobio Co., Del Mar, CA) at 4 °C, and labeled with a fluorescein-conjugated secondary antibody (Molecular Probes, Leiden, The Netherlands). Nuclei were counterstained with DAPI and are seen in blue. Samples were observed with a fluorescence microscope (Olympus, Tokyo, Japan). The number of lymphatic vessels within the tumor was counted in six fields per section: the center regions of the tumor (to measure intra-tumor vessel density) and within an area 1 mm from the tumor border (to measure peri-tumor vessel density). Seven slide sections per mouse were analyzed. To determine the metastasis of SW620 tumor cells to the mouse lateral axillary lymph nodes, lungs, and liver, sections of mouse lymph nodes, lungs, and liver (seven slide sections per mouse) were stained with H&E and rabbit anti-CCR7 monoclonal antibody (Clone: Y59, 1:200 dilution, Abcam Inc. Cambridge, MA).

### Mouse tumor models and *in vivo* procedures

SW620 cancer cells were harvested from subconfluent cell culture plates, washed with PBS, and resuspended at a concentration of 2.5 × 10^7^ cells per ml DMEM containing 10% FBS. Of the suspended cells, an aliquot of 0.2 ml (5 × 10^6^ cells/ mouse) was injected subcutaneously into the right posterior flank of 5-week-old NOD-SCID mice (Orient Company, Korea) with five mice in each group. Three days after tumor cell inoculation, mice were randomized to receive either 125 mg/kg Lithium carbonate (Li_2_CO_3_) in 100 μL deionized water (d.H_2_O) or 100 μL d.H_2_O alone two times per week by oral gavage, from the time of tumor inoculation until the time of humane killing. The serum levels of lithium in treated animals were in the range of 0.7–1.2 mmol/L. We monitored the mice for signs of lithium toxicity, such as weight loss or behavioral changes, and observed none. Tumors were measured with calipers to estimate volumes on days 1 to 62 after injection. Eight weeks after injection, mice were sacrificed and the tumors were resected and weighed. All organs were removed for examination, and metastases to lateral axillary lymph nodes around the tumor region, lungs, and liver were detected by H&E staining and quantified by counting the metastatic lesions in each section. All studies were repeated twice to ensure reproducibility (with a minimum of five mice per group).

### Animal studies

The mice were housed and maintained under sterile conditions in facilities accredited by the Korean Association of Assessment and Accreditation of Laboratory Animal Care. This study was carried out in strict accordance with the recommendations in the Guide for the Care and Use of Laboratory Animals of Yonsei University. The protocol was approved by the Committee on the Ethics of Animal Experiments of the Yonsei University. All efforts were made to minimize animal suffering.

### Western blotting

Cell lysates were fractionated by sodium dodecyl sulfate polyacrylamide gel electrophoresis (SDS-PAGE) and transferred to polyvinyl difluoride membranes. The blocked membranes were incubated with the appropriate antibody, and the immunoreactive bands were visualized with a chemiluminescent reagent as recommended by Amersham Biosciences, Inc., Piscataway, NJ.

### Statistical analysis

All experiments were repeated at least three times. Data are presented as the means ± standard error (S.E.), and statistical comparisons between groups were performed by one-way ANOVA followed by Tukey’s test.

## Additional Information

**How to cite this article**: Maeng, Y.-S. *et al.* Lithium inhibits tumor lymphangiogenesis and metastasis through the inhibition of TGFBIp expression in cancer cells. *Sci. Rep.* **6**, 20739; doi: 10.1038/srep20739 (2016).

## Supplementary Material

Supplementary Information
