# Supplementary material for: Lithium inhibits tumor lymphangiogenesis and metastasis through the inhibition of TGFBIp expression in cancer cells
Source: Sci Rep. 2016 Feb 9;6:20739. doi: 10.1038/srep20739 (PMC4746585; doi:10.1038/srep20739)

Supplementary information

Lithium inhibits tumor lymphangiogenesis and metastasis through the inhibition of TGFBIp expression in cancer cells

Yong-Sun Maeng[1], Seung-il Choi[1], Rina Lee[1], Boram Lee[1], Eung Kweon Kim[1,2]

Figure legends

Supplementary Figure S1 Lithium carbonate inhibits the expression of TGFBIp in colon cancer cells. (**a**) SW620 colon cancer cells were treated with lithium carbonate (125-2000 μM) for 24 h, and cell lysates were subjected to western blot analysis using IgGs against TGFBIp. The membranes were then stripped and re-probed with IgGs against actin to estimate the total protein loaded. (**b**) The mRNA levels of the TGFBIp gene in SW620 colon cancer cells treated with lithium carbonate (250-2000 μM) for 24 h were determined by RT-qPCR. All results were normalized to GAPDH. $^{**}P < 0.01$ vs. control. (**c**) SW620 colon cancer cells were pre-treated with lithium carbonate (250-1000 μM) for 24 h, and then stimulated with TGFβ1 (10 ng/mL) for 8 h. (**d**) SW620 colon cancer cells were treated with lithium carbonate (500-1500 μM) for 24 h. Cell lysates were subjected to western blot analysis using IgGs against TGFBIp, p-GSK3α/β, and p-SMAD3. The membranes were then stripped and re-probed with IgGs against Actin, GSK3α/β, and SMAD3 to estimate the total protein loaded.

Supplementary Figure S2 Schematic diagram of the tumor model and the treatment protocol.

Supplementary Figure S3 Lithium carbonate had no effect on the proliferation of colon cancer cells. SW620 cells (1×10^3 cells) were seeded into a 96-well plate with quadruplicates for each condition. Cells were treated with lithium carbonate (500-1500 μM) for the indicated times. Twenty

microliters of MTT (5 mg/ml) were added to each well and incubated for 4 h. At the end of the incubation, the supernatants were removed, and 150 μl DMSO was added to each well. The absorbance value (OD) of each well was measured at 490 nm. Experiments were performed three times.

Supplementary Figure S4 Lithium-carbonate reduces tumor lymphangiogenesis in colon cancer. Lymphatic vessels in SW620 tumor sections was stained with anti-LYVE1 and hematoxylin and analyzed by microscopy. Arrows indicate lymphatic vessels.

Supplementary Figure S5 Lithium carbonate inhibits the increased migration of LECs caused by TGFBIp in cancer cells. (**a** and **b**) (1, 2) The lower surface of the filter was coated with 10 μg/ml gelatin. LECs (10^5) were seeded onto chemotaxis filters and recombinant human TGFBIp (10 μg/ml) was then added to the lower chamber. After the 12-hour migration period, nonmigrating cells were completely removed from the top surface of the membrane. Migrating cells adhering to the undersurface of the filters were measured by hematoxylin and eosin (H&E) staining and quantified. (3, 4) The lower surface of the filter was coated with 10 μg/ml gelatin. SW620 tumor cells were seeded in the lower chamber to confluence and treated with or without TGFBIp (10 μg/ml). LECs (10^5) were seeded onto chemotaxis filters. (5) The lower surface of the filter was coated with 10 μg/ml gelatin. SW620 tumor cells were seeded in the lower chamber to confluence and treated with lithium carbonate (2000 μM) for 60 min. After a change of media, LECs (10^5) were seeded onto chemotaxis filters. (6) The lower surface of the filter was coated with 10 μg/ml gelatin. SW620 tumor cells were seeded in the lower chamber to confluence and treated with lithium carbonate (2000 μM) for 60 min. After a change of media, TGFBIp (10 μg/ml) was then added to the lower chamber. LECs (10^5) were seeded onto chemotaxis filters. After the 12-hour migration period, non-migrating cells were completely removed from the top surface of the membrane. Migrating cells adhering to the undersurface of the filters were measured by hematoxylin and eosin (H&E) staining and quantified. (**b**)

Migration was quantified. All data are presented as the mean ± SE from three different experiments in duplicate. $**P < 0.01$ *vs*. tumor cell.

Supplementary Figure S6 Lithium carbonate suppresses tumor metastases to lung and liver. (a, b) CCR7 and Hematoxylin staining of frozen sections of lung and liver tissues isolated from mice subcutaneously injected with SW620 tumor cells. Red dotted lines indicate metastatic tumors in the mouse lung or liver.

Supplementary Figure S7 Lithium carbonate suppresses tumor metastasis to the lymph nodes. CCR7 and Hematoxylin staining of frozen sections of lateral axillary lymph node tissues isolated from mice subcutaneously injected with SW620 tumor cells.

Supplementary Figure S8 Schematic diagram of a proposed mechanism to explain the effects of lithium on tumor lymphangiogenesis and metastasis.

Supplementary Figure 1

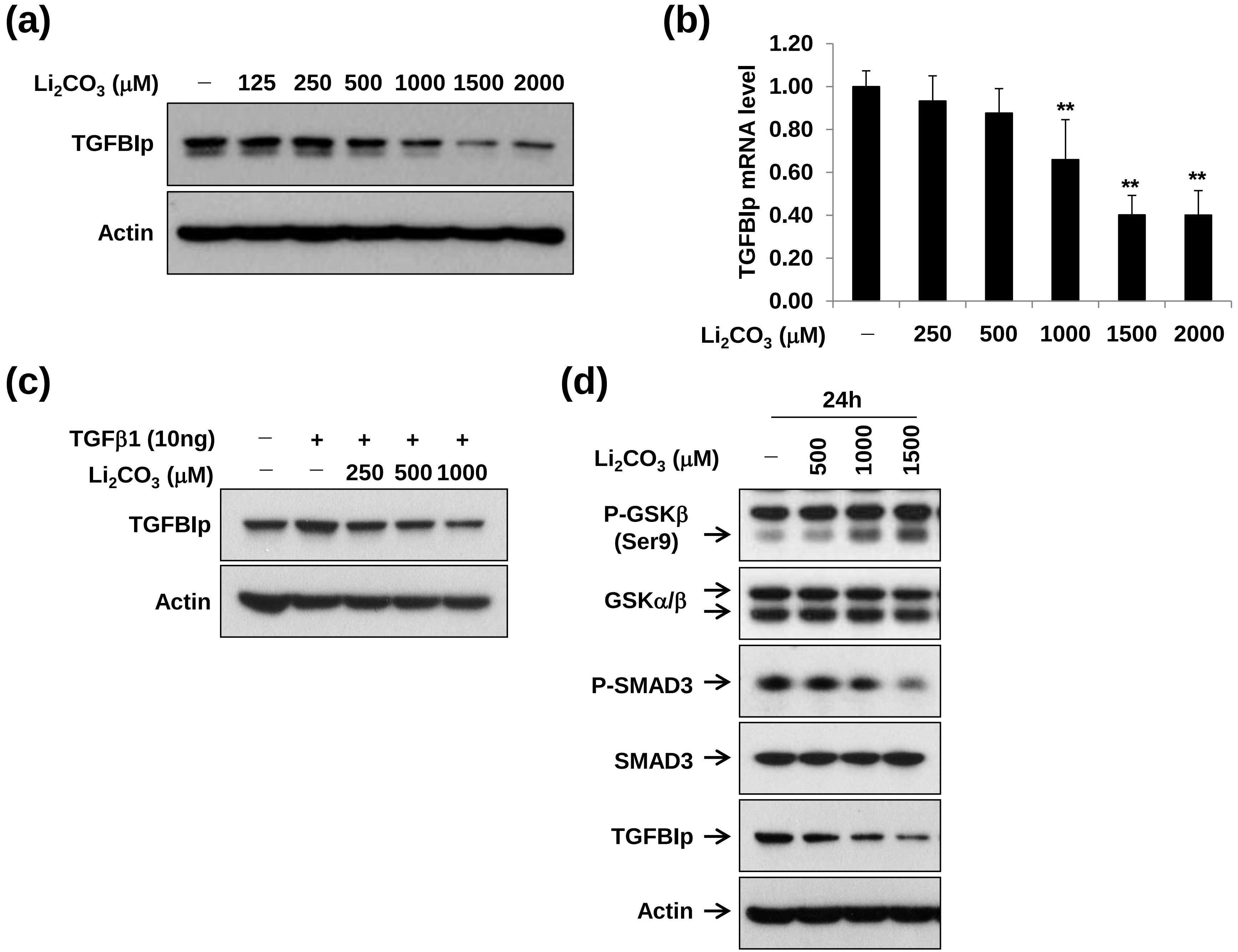

Supplementary Figure 2

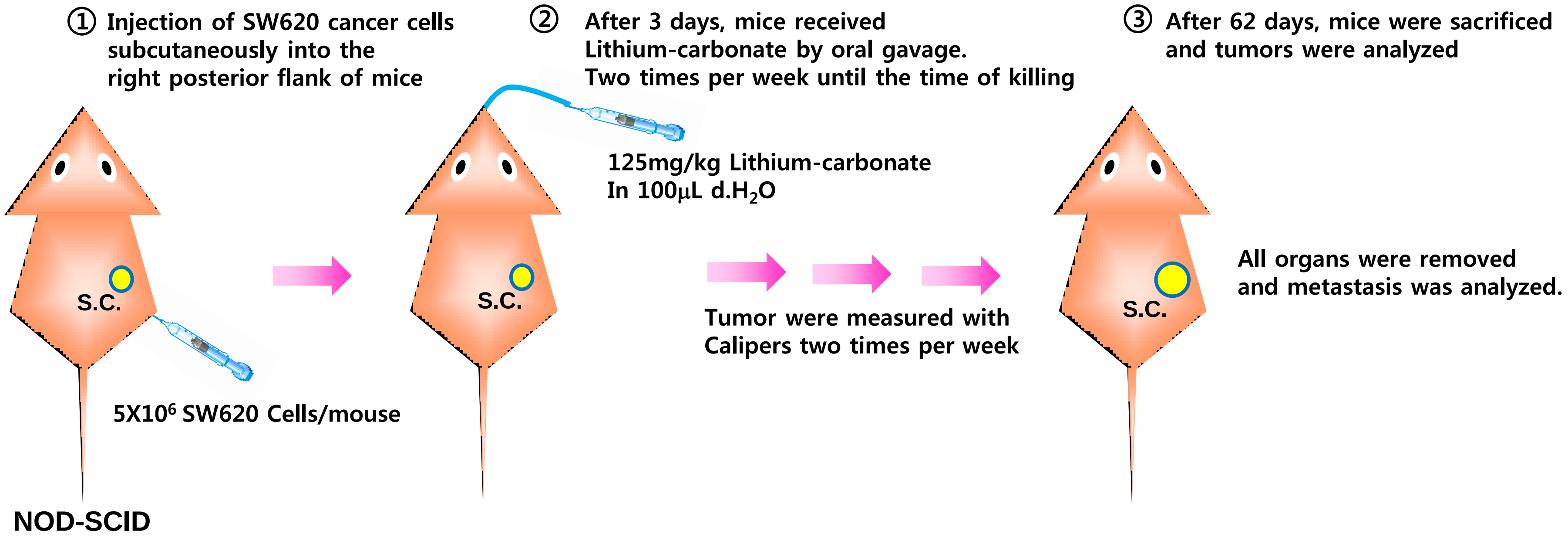

Supplementary Figure 3

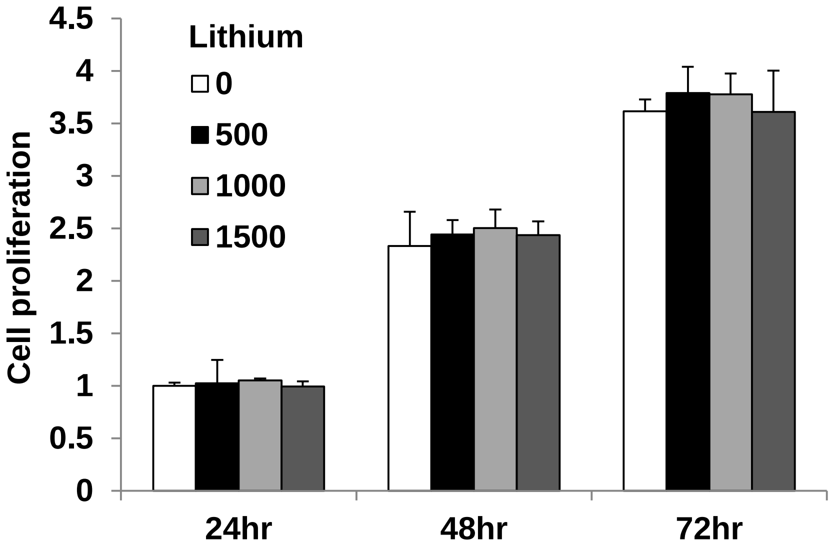

Supplementary Figure 4

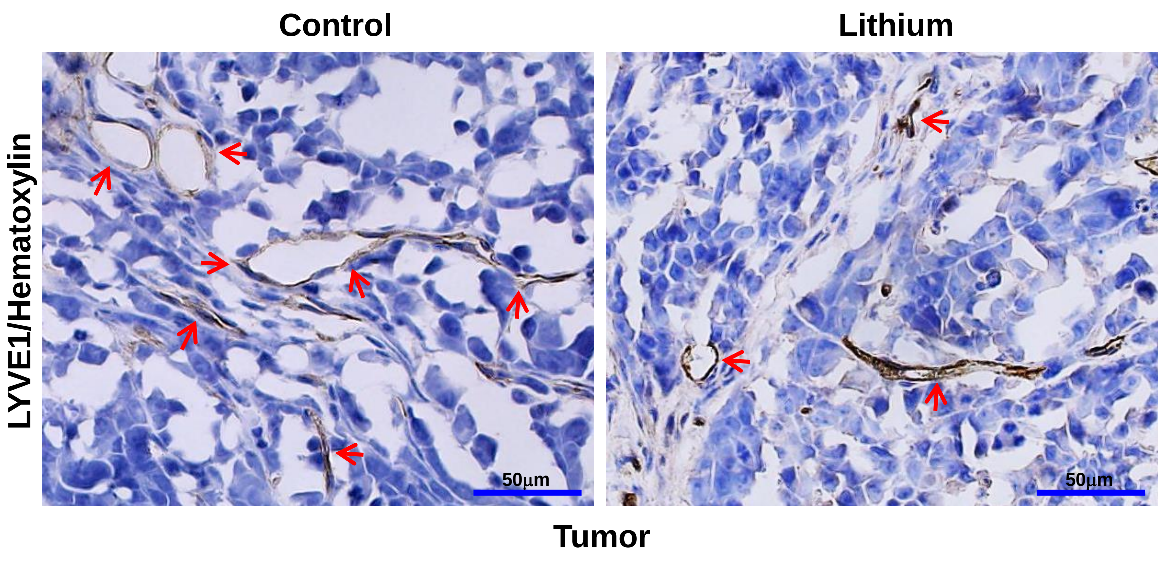

Supplementary Figure 5

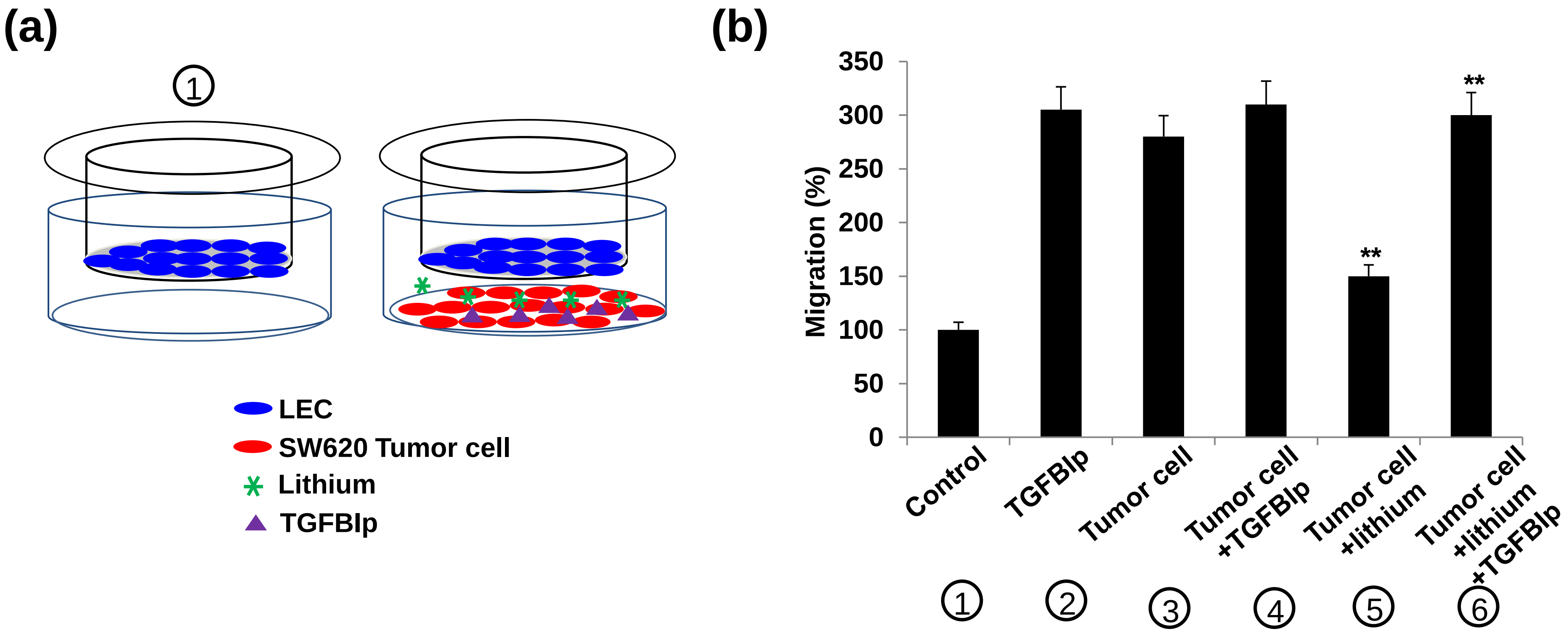

Supplementary Figure 6

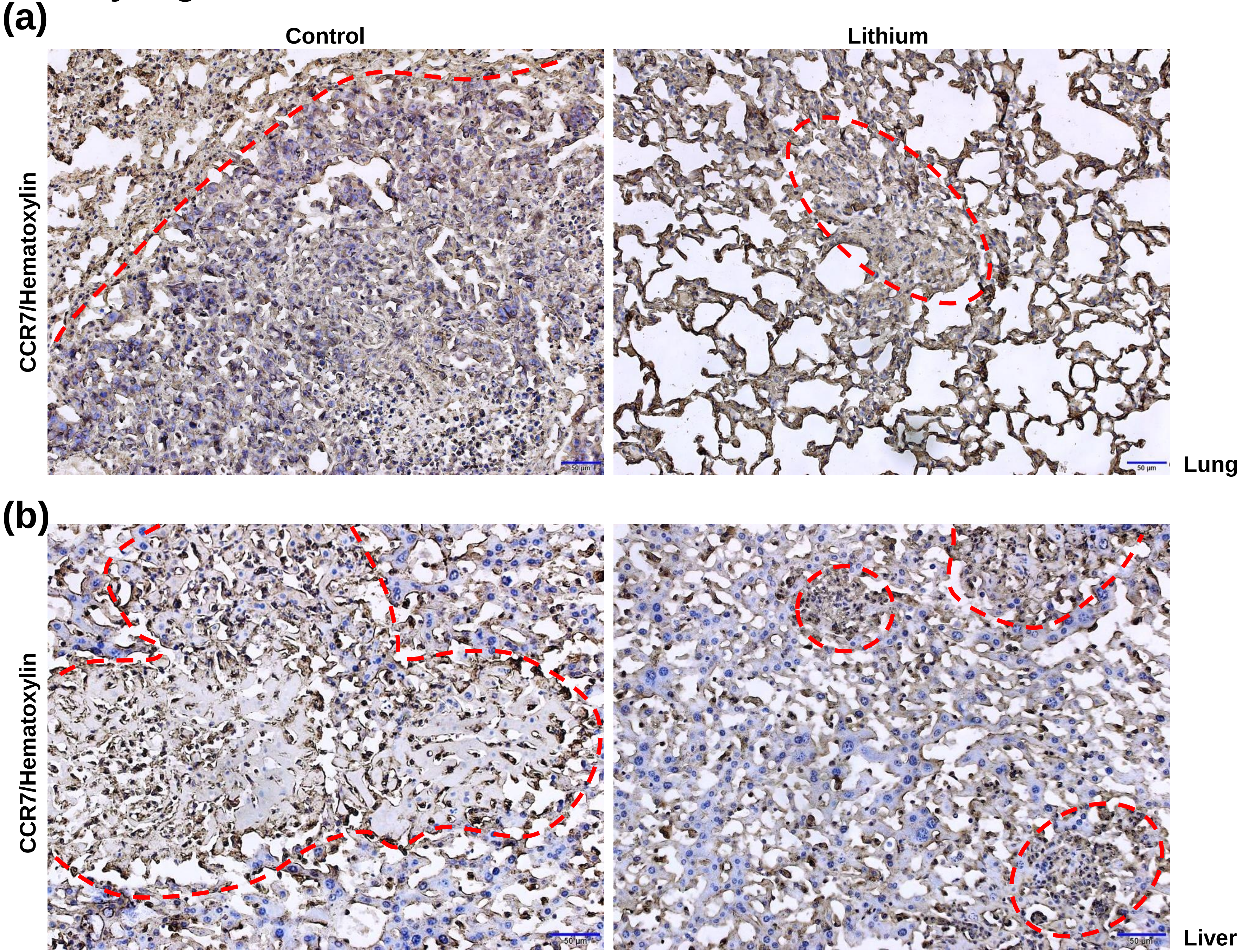

Supplementary Figure 7

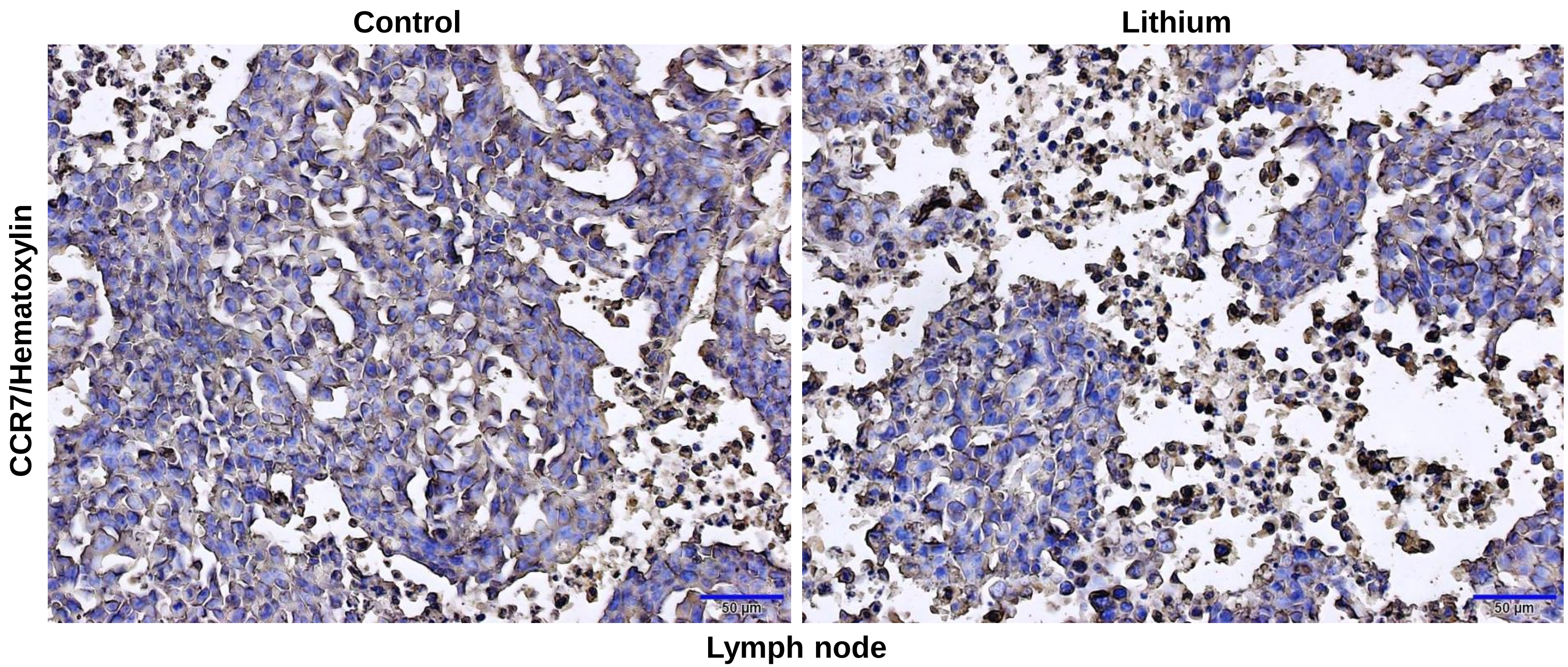

Supplementary Figure 8

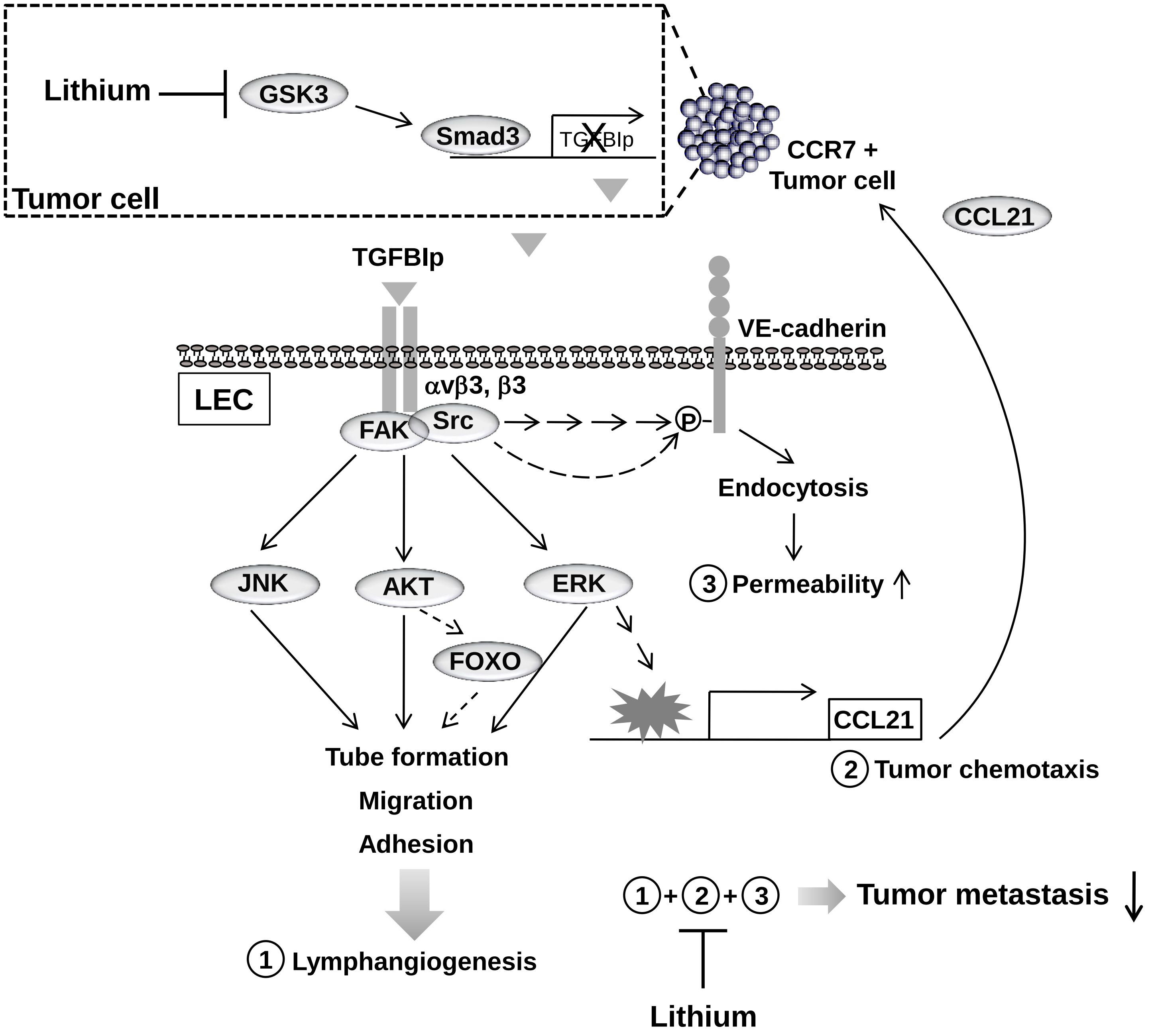

Supplement: Supplementary Information [file srep20739-s1.pdf]
